# Drivers of concentrated predation in an Antarctic marginal-ice-zone food web

**DOI:** 10.1038/s41598-020-63875-y

**Published:** 2020-04-29

**Authors:** Benjamin T. Saenz, David G. Ainley, Kendra L. Daly, Grant Ballard, Erin Conlisk, Megan L. Elrod, Stacy L. Kim

**Affiliations:** 1 College of Marine Science, University of South Florida, St. Petersburg, FL USA; 2 HT Harvey and Associates, Los Gatos, CA USA; 3 Point Blue Conservation Science, Petaluma, CA USA; 4 Moss Landing Marine Laboratories, Moss Landing, CA USA; 5 Present Address: Biota.earth, Berkeley, CA USA; 6 Present Address: Point Blue Conservation Science, Petaluma, CA USA

**Keywords:** Food webs, Ecosystem ecology, Marine biology

## Abstract

Predators impact preyscapes (3-D distribution of forage species) by consuming prey according to their abilities or by altering prey behavior as they avoid being consumed. We elucidate prey (Antarctic silverfish[*Pleuragramma antarctica*] and crystal krill[*Euphausia chrystallorophias*]) responses to predation associated with the marginal ice zone (MIZ) of the McMurdo Sound, Antarctica, polynya. Prey abundance and habitat was sampled across a 30 × 15 km area by remotely-operated vehicle, and included locations that were accessible (ice edge) or inaccessible (solid fast ice) to air-breathing predators. Prey and habitat sampling coincided with bio-logging of Adélie penguins and observations of other air-breathing predators (penguins, seals, and whales), all of which were competing for the same prey. Adélie penguins dived deeper, and more frequently, near the ice edge. Lowered abundance of krill at the ice edge indicated they were depleted or were responding to increased predation and/or higher light levels along the ice edge. Penguin diet shifted increasingly to silverfish from krill during sampling, and was correlated with the arrival of krill-eating whales. Behaviorally-mediated, high trophic transfer characterizes the McMurdo Sound MIZ, and likely other MIZs, warranting more specific consideration in food web models and conservation efforts.

## Introduction

In the marginal ice zone (MIZ) of the Southern Ocean, where sea ice-covered waters transition to open ocean, a combination of both biotic and abiotic habitat features and processes lead to elevated abundance in biological communities relative to adjacent habitat. Melting ice floes create a freshwater lens that increases stratification, reduces surface mixing, and increases light reaching the water column due to decreasing ice cover, all of which encourages blooms of diatoms^1–4^. The MIZ is defined by this melt water lens, extending out on either side of the ice edge. The physical processes determine phytoplankton abundance and species composition, forming the base of the MIZ food web and providing bottom-up influence toward higher trophic levels^5–8^. The zooplankton and micronekton grazers (e.g. krill *Euphausia* spp.)^9,10^ can be abundant in the MIZ, even though their elevated biomass is often maximal beneath the ice cover back from the ice edge^9,11,12^. This indicates that the MIZ may not just be facilitating increased grazer abundance, but may also provide a refuge from air breathing predators under heavier ice cover further into the MIZ. Concurrently, in the outer, open-water portion of the MIZ, predators may have depleted the grazers (see below). In this way, a mid-trophic organism’s location in the MIZ is part of a game of risk, where seeking food must be balanced by predator avoidance^9,11–14^. Part of this game of being consumed or being a consumer is evident at the smaller scale in the patchiness of grazer aggregations and their predators within MIZs^15–19^. Important in that aspect of the game are inter- and intra-specific competition between predators, i.e. a top-down influence. This interaction can negatively affect prey availability where predators overlap their foraging^5,20,21^, if predators are abundant enough to have a measurable impact^13^.

Overall, MIZs constitute a significant Southern Ocean habitat. Antarctica is entirely encircled by a band of sea ice several hundred kilometers wide, its outer circumference constituting the longest MIZ on the planet. This larger-scale MIZ has received appreciable multidisciplinary research attention, with phytoplankton^3,4,9^, grazers^9–12^, and upper trophic levels^22,23^, investigated simultaneously. Within that large-scale band of sea ice, however, occur pools of persistent open water or less concentrated ice, called polynyas. These mesoscale features are found especially along the continental margin and coast^7,8,24^. Around each polynya is also an MIZ that exhibits enhanced productivity and high concentrations of grazer organisms. The trophic importance of polynyas and their MIZs can be further judged by the high densities of top- and mesopredators found there, including seabirds^22,25,26^, seals^27–29^, and whales^20,30^. Another important supporting indicator is the significant association of penguin colonies with polynyas^24,31,32^.

The Southern Ocean MIZs of coastal polynyas have received less of the integrated, multidisciplinary research needed to establish ecological linkages and food web dynamics. Apparent ecological linkages have primarily been inferred, based on coincident enhanced productivity and abundance^1,2,10,20,26,33^. In part, this is a result of logistical difficulties required to make simultaneous observations of predators and the preyscape (3-D distribution of middle trophic levels), along with their immediate environment, especially in the somewhat remote Antarctic. However, recent studies utilizing both established and new technology have directly examined predator foraging in relation to prey availability in comparable areas of the Southern Ocean where sea ice is at least seasonally present^3,4,34,35^. These studies have shown, by inference, the impact of predator abundances and behaviors on small-scale occurrence patterns of prey^5,13,15,19,36^; however the influence of predation and top-down trophic impacts in polynya habitats has not been systematically examined^5^.

**Figure 1:**
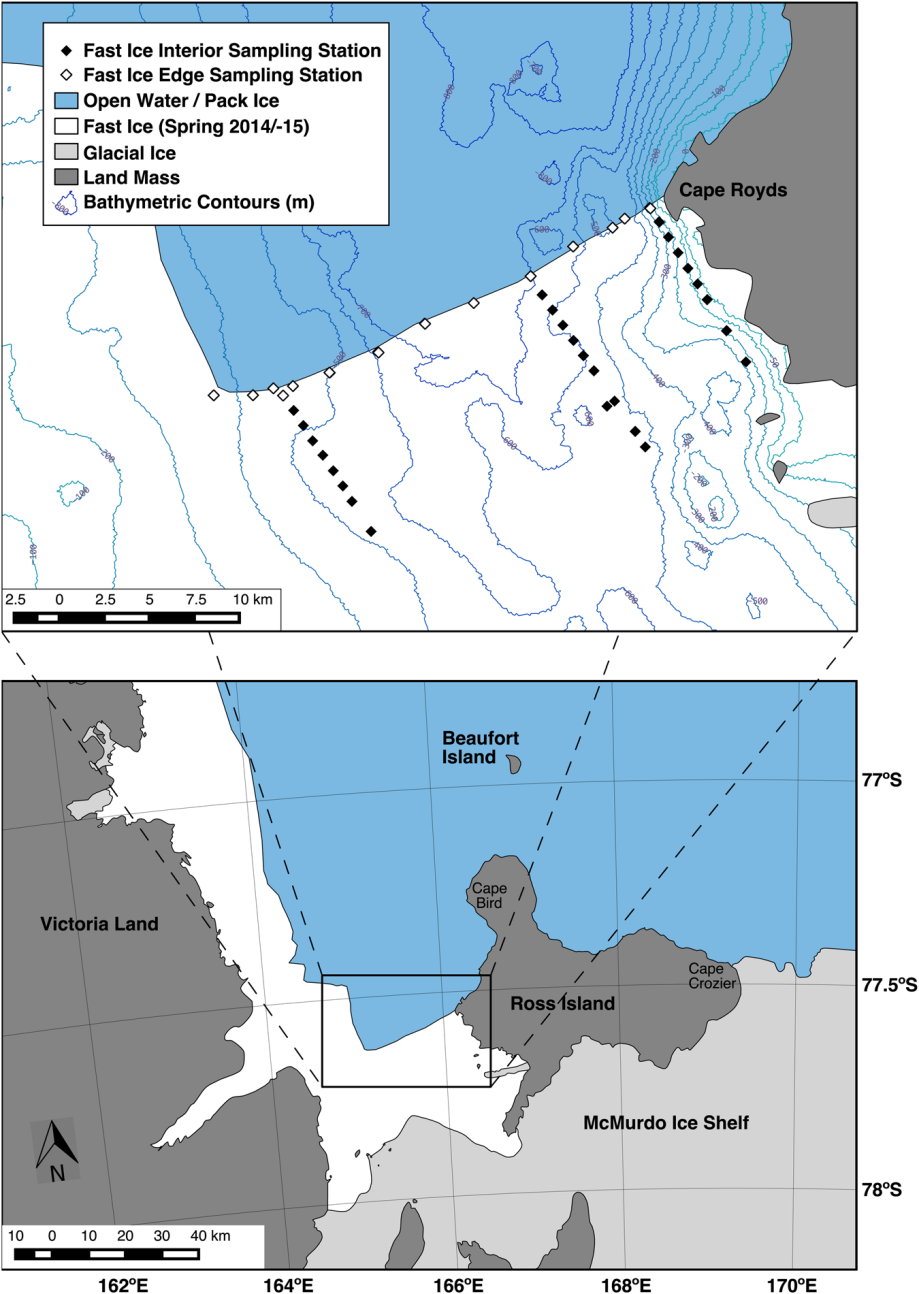
The typical fast ice cover of McMurdo Sound in spring-summer, with the edge extending from Cape Royds across to where it abruptly turns northward (an “L” shape; see inset map). Also shown is the preyscape sampling grid (diamonds), composed of drilled holes through which acoustic and fluorometric sensors were deployed. Adélie penguin colonies occur at capes Royds, Bird, and Crozier, as well as Beaufort Island. Bathymetric contours are at 50 m increments.

**Figure 2:**
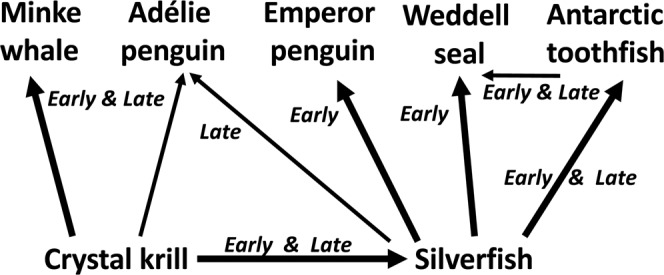
Schematic emphasizing that portion of Ross Sea food web pertinent to the McMurdo Sound MIZ^10,44,45^, and the natural experiment being reported herein. Labeling denotes when in the project’s sampling period each predator is most abundant along ice edge; thick arrows indicate the primary diet or trophic transfer pathway in McMurdo Sound.

In this paper, for the MIZ of the McMurdo Sound, Antarctica, polynya, we address questions of how the preyscape, i.e. 3-D prey availability and forage potential, is influenced by A) animal behaviors, specifically predator avoidance behaviors by the prey, and predation pressure, through resource or interference competition among predators; and by B) physical characteristics of the environment. This MIZ exhibits trophic qualities observed in larger-scale MIZs, including high seasonal primary productivity associated with the polynya and fast ice edge^7,8,24^, and large numbers of predators including penguins^5,21,37^, seals^38,39^, and whales^20,40,41^ (Figs. 1, 2). We synthesize direct prey and predator observations that together constitute a natural predator-exclusion experiment in a before-and-after sequence to quantify the change in prey distribution as a consequence of predation.

## Additional Background

Using what is essentially a natural experiment, we compare the preyscape unavailable to air breathing predators (under the interior fast ice) to the preyscape available to predators in immediately adjacent waters (fast ice edge). The project was organized around a focal predator, the well-studied Adélie penguin (*Pygoscelis adeliae*). While measuring the preyscape, we also measured seasonal change in diet, foraging location and behaviors, and population and breeding dynamics, all of which should contribute importantly to the foraging pressure upon two main prey species, crystal krill (*Euphausia crystallorophias*) and Antarctic silverfish (*Pleuragramma antarctica*)^42–45^. Adélie penguins nesting at Cape Royds, located where the fast ice edge meets Ross Island (Fig. 1), exhibit breeding success equivalent to colonies elsewhere and greater chick growth rates, when the adjacent polynya is open^46,47^. The foraging area of Cape Royds penguins does not expand seasonally as observed at other, larger colonies in the region^37,48,49^, indicating high availability of prey relative to colony size (and thus low total colony foraging pressure). Typically, Adélie penguins at Cape Royds and elsewhere on Ross Island switch from feeding primarily on crystal krill in the early season (~100%) to a diet containing more silverfish later in the season (40–80% fish^5,21,42^; Fig. 2). The switch to more silverfish is closely related to the arrival of whales, especially minke whales (*Balaeoptera bonaerensis*), a baleen species that also feeds on crystal krill^5,21,50^. Prior to the arrival of minke whales, Weddell seals (*Leptonychotes weddellii*) and emperor penguins (*Aptenodytes forsteri*) predominate along the fast ice edge, where they prey heavily on silverfish, 80% and 20–100% of diet, respectively^10,50^.

In this context, we evaluate the extent to which prey availability (krill and silverfish) to penguins in the McMurdo Sound MIZ is driven by physical processes (such as tides, light and sea ice cover), and also by foraging pressure through trophic competition (penguins, whales, seals, and silverfish). Specifically, we hypothesize that predation pressure will alter the preyscape, the predators affecting the availability of their prey by means of their predation (indexed by predators’ abundance along the ice edge). Prey should be depleted where accessible to predators, or prey should occur deeper in the water column, compared to where predators have no access under the ice. In the process, we address the question of why the Adélie penguin diet shifts from one almost entirely of krill to one in which silverfish are prominent, co-incident with the arrival of cetaceans. We provide a 3-D examination of the McMurdo Sound preyscape as it evolved during the spring of 2014–2015.

## Hypotheses Tested

(1) Krill and fish depth distribution corresponds with seasonal phytoplankton distribution within the bloom, i.e. shallow in the water column, to enhance foraging^13^; (2) krill and fish are more prevalent higher in the under-ice water column in the fast ice interior compared to the ice edge, where predators abound^5,12^; (3) Adélie penguins forage where there is reduced light in the MIZ, from shading of sea ice cover^5,51–53^; (4) Adélie penguins from Cape Royds, responding to changing prey distributions, change their foraging strategy (distance from colony and depth of foraging) as the chick-provisioning period progresses^37,48^; and (5) the diet of Adélie penguins changes to one with more fish due to competition upon the arrival of minke whales, i.e. whales are capable of foraging deeper than the penguins and cause a disappearance of krill at shallow depths^5,21^.

## Results

### Preyscape and prey availability

**Figure 3:**
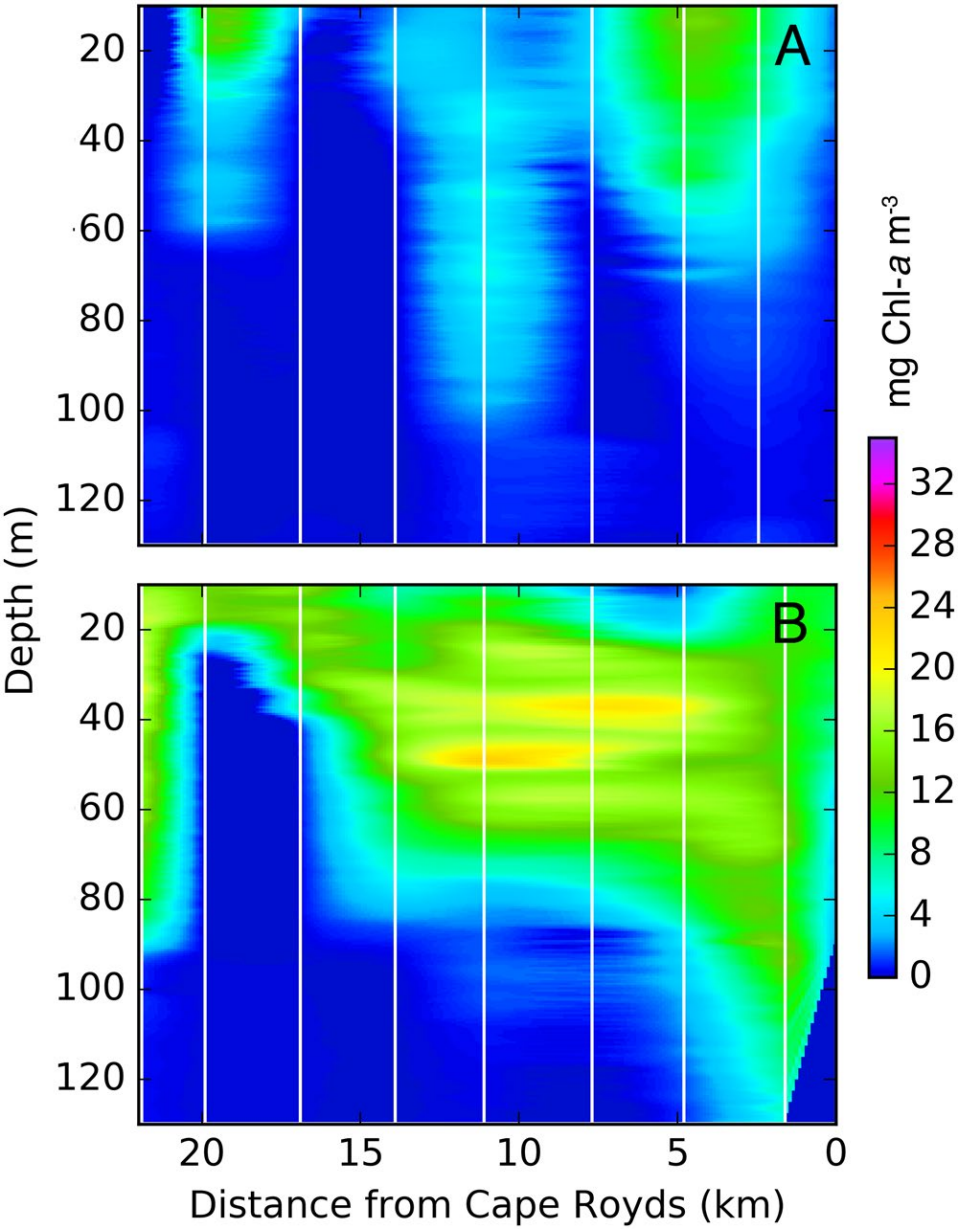
Fluorescence from preyscape sampling at ice edge stations during Pass 2 (**A**; 3–19 December) and Pass 3 (**B**; 20 December – 7 January). Extent of the dense phytoplankton bloom that appeared along the fast ice edge stretched from Cape Royds to the fast ice “L” in the west (Edge stations; Fig. 1). Single, Individual Chl-*a* profiles were located at the white vertical lines within each of the two periods shown, and data were interpolated horizontally.

Phytoplankton concentrations rapidly increased during the first period of sampling (3–19 December; Fig. 3A), and subsequently reached >15 mg Chl-*a* m^−3^ over much of the top 60 m of the water column (after 19 December; Fig. 3B). Despite high variance, extrapolations from fluorescence data indicated that depth of the 1% light level became shallower by pass 3 (20 December-7 January), due to high phytoplankton concentrations, and decreased from a mean of 44 m to 7–22 m for the remainder of fluorescence sampling (until 4 January; Table 1).

**Table 1:** Habitat features and characteristics of foraging by Adélie penguins from the Cape Royds colony, divided into 5–6 d diet-sampling periods, 14 December 2014 to 22 January 2015.

5–6 day diet period	Period start date	Proportion fish in diet	Proportion krill in diet	Mean foraging distance (km)	Mean foraging depth (m)	Mean trip duration (hr)	Minke presence/absence	Depth 1% light (m)
1	14-Dec	7.4 (25.0)	83.9 (36.4)	n.d.	n.d.	n.d.	0	44 (41)
2	20-Dec	19.0 (39.2)	81.0 (39.2)	10.3 (1.3)	47.4 (17.3)	13.3 (5.8)	0	15 (27)
3	25-Dec	33.8 (45.4)	62.3 (46.7)	12.2 (2.0)	52.9 (14.6)	19.1 (8.6)	1	7 (3)
4	30-Dec	45.2 (49.8)	54.8 (49.8)	11.9 (3.4)	49.5 (17.1)	16.2 (6.8)	1	22 (28)
5	4-Jan	50.0 (52.2)	50.0 (52.2)	11.9 (5.0)	46.7 (21.1)	16.9 (9.4)	1	12 (5)
6	9-Jan	55.9 (44.1)	44.1 (44.1)	10.9 (6.2)	54.0 (18.6)	26.7 (12.2)	1	n.d.
7	14-Jan	47.9 (43.2)	52.1 (52.1)	10.8 (8.3)	50.6 (18.3)	12.0 (4.3)	1	n.d.
8	19-Jan	44.5 (51.3)	55.5 (51.2)	n.d.	n.d.	n.d.	1	n.d.

Periods are identified as the first date of a 5–6 d span. “No data” is abbreviated as “n.d.” Standard deviations are given in parentheses.

Over all fast ice sampling stations, including interior and edge, silverfish observations became severely reduced in the upper 200 m of the water column over time; in linear terms, the energy backscatter from silverfish was 17 times greater before than after 24 December. In contrast, the linear backscatter energy from krill aggregations increased, and was 28 times higher during the last preyscape sampling period compared to the first.

Among interior fast ice stations (Fig. 1), silverfish detections were reduced after the first sampling period (mean acoustic backscatter decreased below −80 dB; Table 2). The average detection depth of silverfish was 80–100 m. In contrast, krill detection at interior stations increased over time (from a low of −88.7 dB before 20 December, to a high of −73.0 dB after 4 January; Table 2), and their mean detection depth decreased (37.4 m before 20 December to 24.6 m after 4 January). At ice edge stations, silverfish aggregations were detected during two of the five sampling periods, while krill were detected more frequently. We could not infer separate trends over time in silverfish or krill abundance or depth distribution at the ice edge due to the smaller number of stations (Fig. 1) and patchiness of the acoustic detections.

**Table 2:** Characteristics of the contribution to the preyscape by crystal krill and Antarctic silverfish, with sampling passes two and three divided into shorter periods.

	Fast Ice Interior Stations				Fast Ice Edge Stations			
	Mean silverfish S_v_	Mean silverfish depth, m	Mean krill S_v_	Mean krill depth, m	Mean silverfish S_v_	Mean silverfish depth, m	Mean krill S_v_	Mean krill depth, m
1 (14 Dec)	−80.2 (−80.4, −80.0)	81.9 (80.1, 83.6)	−88.7 (−89.8, −87.7)	37.4 (33.3, 41.9)	−80.6 (−81.6, −79.5)	84.8 (74.4, 93.8)	−81.8 (−81.9, −81.7)	91.4 (90.7, 92.0)
2 (20 Dec)	−98.4 (−99.7, −97.1)	16.3 (13.4, 19.3)	−82.0 (−82.3, −81.6)	32.6 (29.4, 37.5)	none	none	−80.6 (−80.8, −80.4)	89.8 (89.6, 90.1)
3 (25 Dec)	−92.6 (−92.7, −92.4)	126 (124, 127)	−75.1 (−75.2, −75.0)	29.0 (28.8, 29.2)	none	none	−88.3 (−88.6, −87.9)	24.9 (24.0, 26.2)
4 (30 Dec)	−94.3 (−94.7, −93.9)	77.5 (73.7, 81.2)	−93.8 (−94.2, −93.4)	58.6 (55.3, 61.6)	−95.0 (−95.5, −94.5)	25.2 (24.8, 25.7)	−98.4 (−99.5, −97.4)	23.0 (21.9, 24.2)
5 (4 Jan)	none	none	−73.0 (−73.3, −72.6)	24.6 (23.1, 26.0)	none	none	−78.4 (−78.6, −78.1)	50.1 (49.1, 51.1)

Boostrap-calculated 95% confidence internals (10000 replications) are shown in parentheses. S_v_ = mean acoustic volume backscattering strength (dB re m^−1^; smaller values (e.g., −100) indicate weaker backscatter than larger values (e.g., −83).

**Figure 4:**
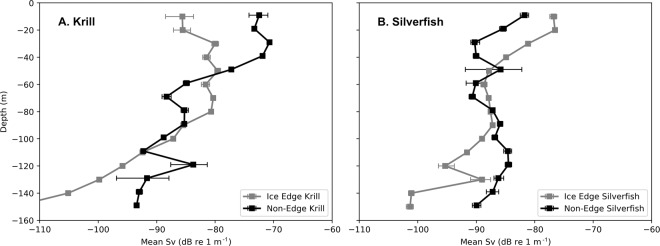
Depth distribution of krill and silverfish at fast ice edge vs fast ice interior (non-edge) stations during sampling passes 2 and 3. Values are mean Sv (acoustic backscatter dB re 1 m^−1^) by 10-m depth bins; smaller values indicate weaker backscatter than higher values. Note that Sv values indicate relative abundance within taxa, but do not measure relative biomass of krill compared to silverfish. Error bars indicate the bootstrap-calculated (10000 iterations) 95% confidence interval of the mean.

Permutation tests indicated that ice edge stations had lower krill abundance compared to interior locations, at depths of 10–40 m (Table 3, Fig. 4). In contrast, detected silverfish aggregations had largely the opposite pattern, with more silverfish found at the fast ice edge compared to the interior at 10–30 m depth.

**Table 3:** Permutation test results evaluating acoustic backscatter from krill and silverfish, at ice edge and interior as affected by depth, 14 December 2014 to 7 January 2015.

Depth Interval	Krill		Silverfish	
	p-value	location of greater backscatter	p-value	location of greater backscatter
0–10 m	0.19	—	0.16	—
10–20 m	0.04**	interior	0.06*	edge
20–30 m	0.04**	interior	0.04**	edge
30–40 m	0.07*	interior	0.13	—
40–50 m	0.48	—	0.43	—
50–60 m	0.21	—	0.65	—
60–70 m	0.05*	edge	0.77	—
70–80 m	0.19	—	0.58	—
80–90 m	0.42	—	0.58	—
90–100 m	0.40	—	0.56	—

Shown are p-values found using eqn. 1 (Methods), where an even distribution of prey would produce a value of 0.5. Significance at the 95% level is indicated by **, and marginal significance at the 90% level is indicated by *. Edge and Interior locations indicate sampling from stations found along the fast ice edge or interior (>1 km from the fast ice edge), respectively (Fig. 1). Comparisons below 100 m were not calculated due to declining detectability with depth, and high variance of abundance.

### Penguin foraging in the marginal ice zone

**Figure 5:**
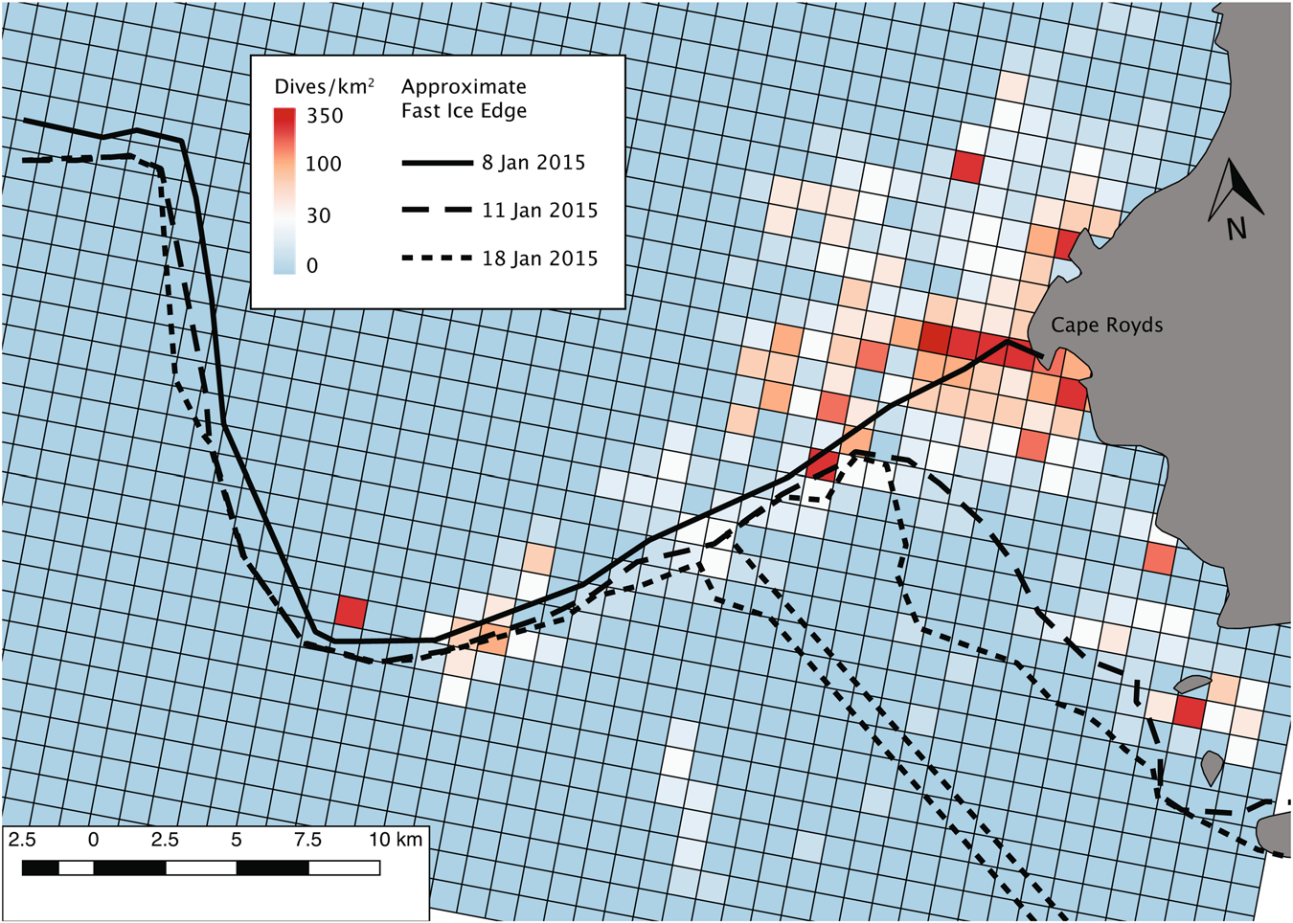
Locations of foraging dives of tagged Adélie penguins nesting at Cape Royds, 20 December 2014–13 January 2015. Penguin foraging activity southward of the 8 January ice edge shows penguins tracking its southward retreat. Dives southward of the 18 January edge (i.e. several cells inside the ice edge) are likely due to erroneous dive geolocation, which may contain inaccuracies of a few kilometers.

Satellite-tagged penguins foraged within ~15 km of the Cape Royds colony; mean penguin foraging trip distance among tagged penguins ranged 10.3–11.9 km. Foraging was more concentrated along the fast ice edge (Figs. 1, 5), than in more open water and pack ice farther north. Minke whales also foraged along the edge^42^. In early January, the pack ice was blown north, leaving mostly open water along the fast ice edge; sequential break out of the fast ice followed, especially in the eastern Sound near the penguin colony^54^. As the fast ice broke away, penguins (and whales) foraged in the leads, following the southward-receding fast ice edge (Fig. 5). As judged from instrumented penguins, the greatest number of foraging dives were made within about 2 km of the ice edge. Greater accuracy was not possible in satellite-derived assessment of the fast ice edge position because of the intermittent presence of dense pack ice, which was indistinguishable from fast ice in the satellite imagery.

**Figure 6:**
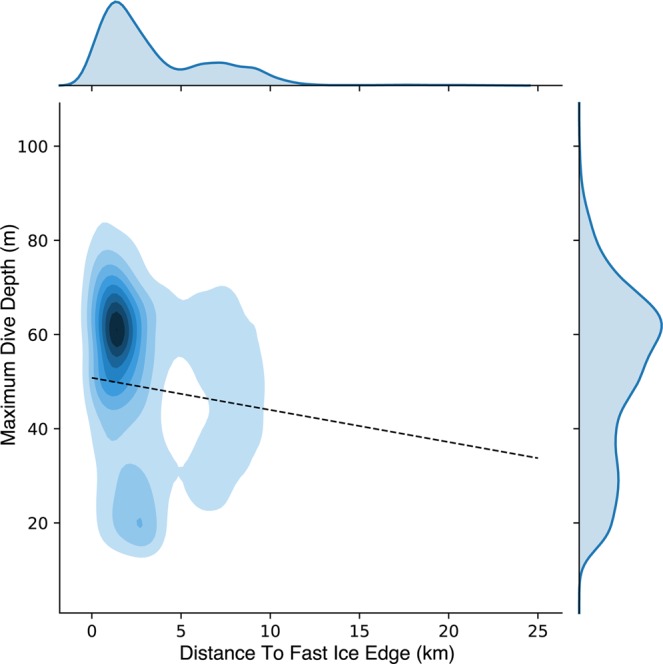
Kernel density plot of penguin foraging dives comparing maximum dive depth to the distance to the fast ice edge. The significant fixed-effect model predicting maximum dive depth is shown as a dashed line. Kernel density contours show deeper dives within 5 km of the edge.

Maximum dive depths were positively related to proximity to the fast ice edge (Table 4). As penguins foraged farther north into the polynya (up to 20 km from ice edge), maximum dive depths grew shallower by up to 14 m (Fig. 6). In addition, the number of undulations per dive (indicating prey capture behavior) decreased significantly with increasing distance from the edge (Table 4), equivalent to drop of ~2 per dive between the ice edge and 20 km distant, a drop of 13% from the mean of 15.7. Diving behavior clustered around deeper diving depths (~60 m) within 5 km of the edge (Fig. 6). Beyond 5 km, foraging dives had more evenly distributed maximum depths. There was also a smaller, secondary cluster of diving with a peak centered at 20 m depth within 5 km of the ice edge (Fig. 6).

**Table 4:** Regression parameters for the fixed-effect of distance to the fast ice edge in mixed-effects models of Adélie penguin dive characteristics.

Criterion	Mean (S.D.)	Coefficient (S.E.)	Z	P > | Z |	Marginal r^2^	Random Intercept Variance (S.D.)	Random Residual Variance (S.D.)	Within ID Correlation
Mean light at dive bottom phase	2.8 (1.4) lux	0.035 (0.003)	11.899	<0.001	0.014	0.552 (0.743)	0.427 (0.653)	−0.044
Mean light at 5 m depth	152 (150) lux	0.000 (0.002)	−0.014	1	—	0.061 (0.248)	0.197 (0.444)	−0.089
Maximum Dive Depth	51 (18) m	−0.682 (0.069)	−9.930	<0.001	0.014	150.7 (12.3)	236.7 (15.4)	−0.062
Dive undulations	15.7 (7.4)	−0.098 (0.028)	−3.506	<0.001	0.002	17.0 (4.12)	38.5 (6.21)	−0.075

**Figure 7:**
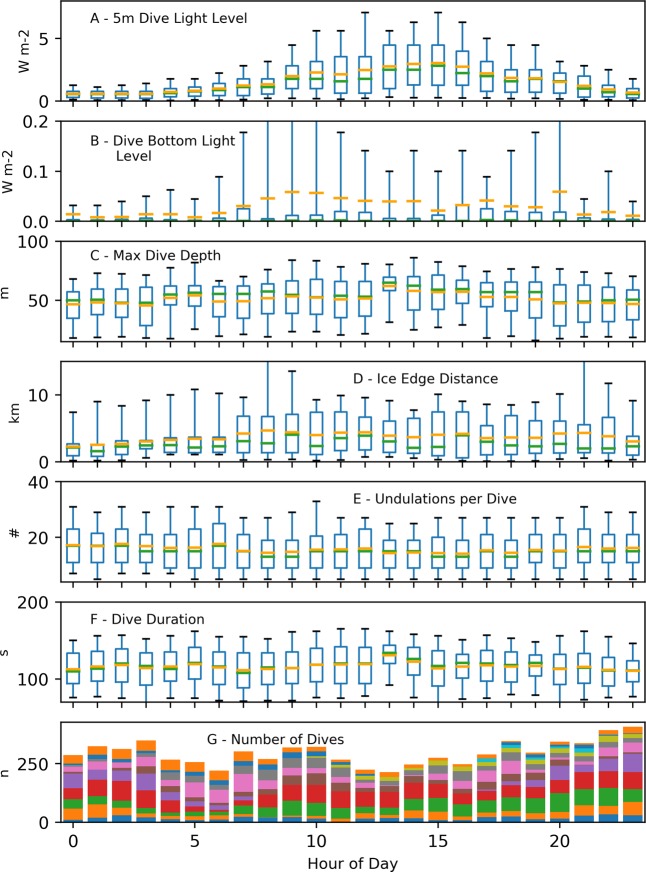
Boxplots of hourly variability in penguin diving parameters. Box top and bottom boundaries show upper and lower quartiles of the dive parameters, while the whiskers represent the 5^th^ and 95^th^ percentile of the parameter. Green and orange lines show the median and mean, respectively of the hourly dive parameters. The different colors in the stacked bar plot (**G**) correspond to the number of dives recorded from individual penguins. Maximum diving depth (**C**), proximity to the ice edge (**D**), and dive undulations (**F**) varied according to a daily sinusoid, and corresponding to light availability at 5 m depth (**A**), and light at the bottom of dives (**B**). Dive duration (**F**), and the number of foraging dives attempted (**G**), showed significant correlations also, but the model effects were inconsequential.

Daily, penguin dive behaviors were significantly correlated with a 24 h period sinusoid (Table 5). Near-surface light (at 5 m) during foraging dives varied sinusoidally across the day, following idealized solar availability at ~77° S, and bottom light during foraging dives followed the same pattern with more intense light found on dives closer to local noon (Fig. 7). Penguins made slightly deeper dives (~2.5 m deeper; Table 5) when more light was available during the middle of the day, but fewer undulations occurred per dive during this period (~2 undulations). Interestingly, penguins dove ~2 km farther away (generally northward) from the ice edge during mid-day (Fig. 7). Neither dive duration, nor the number of foraging dives had meaningful model effects (i.e. small coefficients), although the models showed significant correlations (Table 5).

**Table 5:** Regression parameters for the fixed-effect of daily sinusoidal variation in mixed-effects models of Adélie penguin dive characteristics.

Criterion	Coefficient (S.E.)	T	P > | T |	Marginal r^2^	Random Intercept Variance (S.D.)	Random Residual Variance (S.D.)	Within ID Correlation
*Mean light at 5 m depth	0.282 (0.007)	39.8	<0.001	0.16	0.043 (0.207)	0.161 (0.401)	0.010
*Mean light at dive bottom phase	0.142 (0.012)	12.4	<0.001	0.01	0.537 (0.732)	0.426 (0.653)	0.005
Maximum Dive Depth	1.377 (0.273)	5.0	<0.001	0.00	134.3 (11.59)	239.2 (15.46)	0.007
*Distance to fast ice edge	0.166 (0.017)	9.7	<0.001	0.01	0.456 (0.676)	0.941 (0.970)	0.007
*Dive undulations	−0.052 (0.007)	−6.9	<0.001	0.01	0.075 (0.274)	0.180 (0.424)	0.008
Dive duration	−0.885 (0.374	−2.4	0.02	0.00	212 (14.6)	448 (21.1)	0.007
Number of dives	−0.068 (0.012)	5.3	<0.001	0.00	0.048 (0.204)	—	0.019

*Log-transformed criterion variable.

The average maximum depths of penguin foraging ranged 47.4–54.0 m (Tables 1, 4) during 5–6 d periods, with an overall mean of 50.2 ± 0.25 SE m. Foraging trip duration averaged 17.3 ± 1.6 SE hr. The shortest durations occurred at the start of chick feeding and the longest occurred near the end, but we did not detect a linear trend (range of average per 5 d diet period, 13.3–26.7 hr; Tables 1, 4). Neither did we detect trends across time among the diving light levels, dive bottom time, or undulations per dive during the ~5 weeks in which penguin foraging was investigated.

### Prevalence of penguin competitors

**Figure 8:**
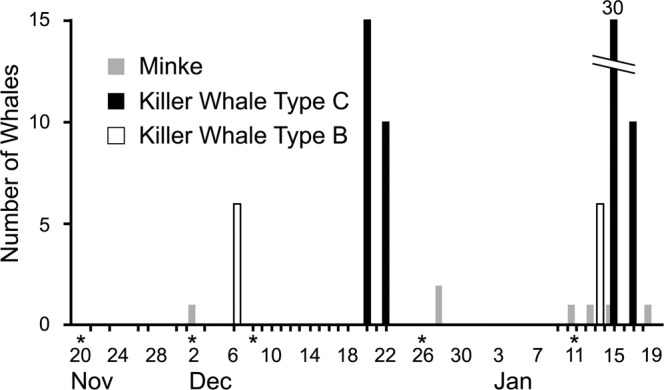
Observations of cetaceans at Cape Royds. Data acquired by using binoculars from a 30 m high, coastal hill, once or twice per day weather permitting, 20 November 2014–18 January 2015. X-axis ticks indicate days when observations were made; asterisk (*) indicates dates with verification by helicopter survey.

Potential air-breathing competitors to Adélie penguins observed during the study included whales, Weddell seals, and emperor penguins. The first fish-eating, type-C killer whales were seen from Cape Royds on 20 December, and were observed sporadically thereafter (Fig. 8), their presence being confirmed by helicopter flights along the ice edge. During those flights ≥ 100 individuals were seen regularly in the vicinity of the ice edge “L” (beyond observation distance from Cape Royds; Figs. 1, 5) after mid-December. The first krill-eating minke whale was seen 2 December, but they were not seen regularly until after 28 December, often from Royds (Fig. 8). Ecotype B (mammal-eating) killer whales were seen irregularly after 5 December, numbering at least 6 individuals (for more details see^20^).

**Figure 9:**
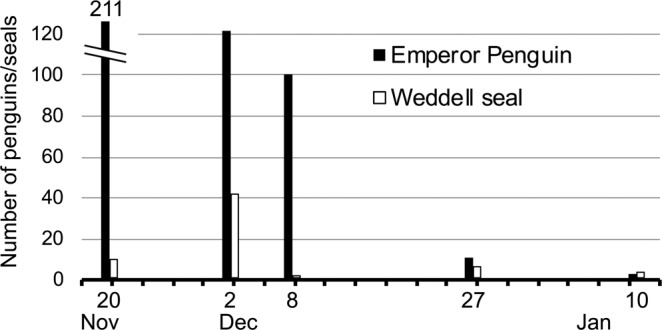
Numbers of silverfish-eating emperor penguins and Weddell seals hauled out along the McMurdo Sound fast ice edge. Counted by helicopter, 2014–15.

Helicopter surveys along the fast ice edge indicated 211 fish-eating emperor penguins were present during the first flight in mid-November (Fig. 9). That number rapidly decreased until very few were seen after the first week of December, when these penguins left the study area to molt. Similarly, fish-eating Weddell seals were present in November but disappeared in early December along the fast ice edge; more than 1,000 remained present in the interior of the fast ice, spreading out from their main eastern Sound breeding area after the last week of November^37^.

### Relationship between penguin diet, preyscape, and competitors

**Figure 10:**
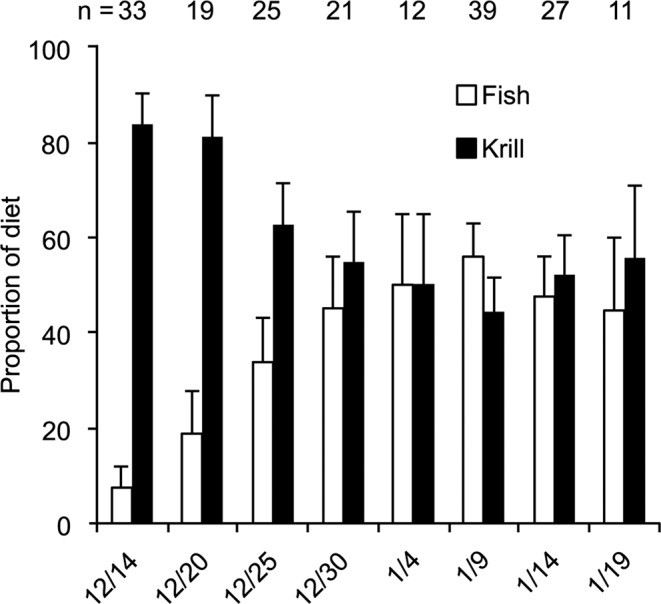
The average composition of the diet fed to chicks beginning when ~5% of the nests contained chicks. Values grouped into 5–6 d intervals (identified by initial date) centered to correspond with the prey sampling. Numbers along the top indicate sample size of parental feeds observed; error bar is the period standard deviation of the diet component.

Consistent with observations made in previous years at Cape Royds^21,42,43,46^, at the start of the 2014 penguin chick rearing period (mid-December), crystal krill dominated penguin diet, but over the next two weeks, 14 to 30 December, the proportional composition of the diet included more Antarctic silverfish (Fig. 10; Table 1). Once krill and fish contributed equally to the diet, that ratio continued over the following four weeks.

To test for relationships between diet and the preyscape, data were assembled into 5–6 d period means (Table 1), complementing the prey availability data (Table 2). Emperor penguins and Weddell seals were decreasing rapidly along the ice edge during the start of Adélie penguin biologging. Since killer whale and minke whale presence corresponded temporally, we included a single presence/absence variable to represent whales, both species, as a potential factor in penguin diet change (Table 6; also^5,21^). As a proxy for water clarity, which could potentially influence diet by affecting the penguins’ ability to locate and pursue prey, we included mean depths of 1% light level calculated from fluorescence profiles using a model of light transmission (Fig. 3). The penguin foraging characteristics tested included period means of foraging trip distance and maximum dive depths (as a potential difference in prey prevalence by depth). Amongst these preyscape variables (shown in Tables 1–3), the only variable that had any effect on change in penguin diet was the presence of whales (Table 6).

**Table 6:** Correlations between preyscape variables and the fractional contribution of krill to the penguin diet (Holm-Bonferroni-corrected). Asterisk (*) significant p-value at the 5% alpha level.

	Kendall tau	p-value		n observations (periods)
Foraging depth	0	1	6	
Foraging distance	0	1	6	
Foraging duration	0.2	0.598	6	
Silverfish backscatter, all	0.333	0.505	5	
Silverfish depth, all	0	1	4	
Krill backscatter, all	−0.4	0.332	5	
Krill depth, all	0.527	0.213	5	
Krill backscatter, edge	0	1	5	
Krill depth, edge	0.6	0.149	5	
Depth 1% light	0.4	0.332	5	
Whale presence	−0.655	0.046*	8	

## Discussion

The results presented above, based on a natural experiment, represent an effort to quantify seasonal changes in the preyscape of the McMurdo Sound Polynya MIZ, and to understand what processes were responsible for the changes. We have described several relationships, some of which have been inferred or described previously but not simultaneously, that relate specific features of the MIZ to prey abundance and distribution, and predator activity. Taken together, we interpret these results as support for behavioral, or top-down, forcing as a mediator of trophic interactions. In particular, decreased krill abundance in the upper water column, and the prediction of penguin diet change by whale presence, indicate that prey and predator behavior alter the preyscape, within the context of the McMurdo Sound ice edge.

Reduced krill abundance in the near surface layers (10–40 m depth) at the fast ice edge corresponds to where penguins were foraging, overlapping the average recorded dive profiles of penguins (average maximum depths on foraging dives ranged 47–54 m). Simultaneously, minke whales were foraging persistently and on average to 72 m (range 19–130 m)^41^. Thus contracting krill abundance could be due to local depletion by predation, or because of predation avoidance behavior by krill (moving either deeper or further under the fast ice). Either case constitutes a behavioral modification of prey availability. In a mini-natural experiment evident in the 2019–20 summer, the Cape Royds penguins had exhibited their shift from krill to fish with arrival of whales (n = 80 diet observations, 13 December-7 January, eventually 65% fish). Then, when a 2 km wide band of fast ice (5 km along the outer edge, adjacent to Cape Royds) suddenly broke away, penguin diet switched immediately to 69% krill (n = 22, 8–12 January), followed by a switch back to fish (n = 88%, 15–20 January) with arrival of more minke whales (DGA, pers. obs.). As demonstrated in the present project results, it appears that the krill had been avoiding being too close or had become depleted near the fast ice edge.

Given the large vertical extent of the phytoplankton bloom observed during the study, it is likely that conditions were optimal for krill foraging in terms of food resources. Michaelis-Menton curves fitted to Antarctic krill (*E. superba*) growth (length) compared to environmental Chl-*a* levels show half-saturation constants of just 0.33–0.5 µg Chl-*a* L^−1^ ^55-57^; insofar as crystal krill (a smaller krill species) growth saturation rates are not appreciably faster, Chl-*a* concentrations should have been saturating for crystal krill over at least the top 100 m of the water column during our study (Fig. 3). Given that food resources were sufficient down to 100 m, krill would be expected to reduce their high risk of predation in shallower waters by moving deeper in the water column or under the fast ice. While observed krill distributions match this risk-reward theory (confirming Hypothesis 2), we did not observe any locations that could be classified as unsuitable for krill foraging in the vertical dimension. Therefore we were not able to precisely evaluate whether krill distributions where associated with their foraging resources (Hypothesis 1). The vertical distributions of silverfish aggregation patterns likewise could not be effectively compared to the phytoplankton bloom due to its almost complete overlap with our acoustic observation range.

Following from the risk-reward theory, if foraging opportunities for krill were not restricting, then other factors besides risky prey behavior made the McMurdo MIZ a hotspot for penguin and whale foraging during our study period. Heavy pack ice cover associated with the fast ice edge, a stark boundary of both light availability and predator exclusion, may lead to greater prey prevalence nearer the surface, given the light-avoidance behavior exhibited by both krill and silverfish to avoid visual predation^52,58,59^. Marine predators often aggregate at frontal zones where habitat changes abruptly^22,60^, where they take advantage of prey species’ transitional behaviors as they move between environmental conditions^61^. Confirming Hypothesis 3, penguins in this study made deeper, darker dives and performed more undulations (indicative of prey capture) while concentrating foraging at the McMurdo Sound fast ice edge, similar to penguins in the Ross Sea that apparently encounter more prey during darker dives^62^. Typically snow-covered, McMurdo Sound fast ice transmits <1% of incoming solar irradiance^63,64^ and underneath the fast ice, light-avoiding nekton appear to shoal^5,52^. Any shallow prey that migrate away from the cover of sea ice into the brighter lightscape of the MIZ may experience an increased risk of predation and consequently provide increased foraging opportunity for visual predators, such as fish^59,65^ and air-breathing predators.

Cape Royds penguins during 2014–15 did not dive progressively deeper over the chick-feeding season, which is counter to the pattern observed at the nearby, but much larger, Cape Crozier penguin colony^5,21^. Instead, the average maximum foraging depth remained at ~50 m. This is the same depth at which Crozier penguins began foraging, with increasingly deeper dives as the chick feeding period progressed. Foraging consistently within 20 km of the colony, instrumented penguins at Royds did not need to travel far to find food. Hypothesis 4, that penguins would change foraging range and diving behavior with changing prey resources, was not supported as their behaviors indicate penguins were prey-replete, despite reduced availability of shallow krill at the ice edge. Prey species were detected at 50% of sampling stations before 20 December and at 100% of the stations afterward. In waters of the MIZ of the Ross Sea Polynya (the other side of Ross Island, off Cape Crozier), where the preyscape was quantified in 2012–13, penguins foraged to ~50 m depth early, and increased to 80 m later^5^. Note that Adélie penguins have reached neutral buoyancy near 50 m, at and below which energy expenditure would be minimized^66^. At Cape Crozier, three orders of magnitude more penguins, plus competition from minke whales^5,21^, apparently depleted krill in the upper water column near to the colony, necessitating that penguins dive deeper and travel farther later in the season; with deeper dives they brought back more energy-rich fish^5^. The predation from minke whales and the relatively small number of penguins at Cape Royds did not result in inter-seasonal shifts in penguin foraging depth or distance.

Perhaps related to the apparent prey saturation, the prediction of Hypothesis 5, that prey availability related to competition would be a determinant in penguin diet, was not clear for Cape Royds penguins during this study. This hypothesis was derived from the larger Cape Crozier colony, where both very abundant penguins as well as minke whales foraged resulting in local prey depletion and apparent exploitive inter- and intraspecific competition^5,21^. The penguin diet at Cape Royds similarly shifted to include more fish over time, however the relative abundance of crystal krill increased and silverfish aggregations, insofar as we could measure their availability, decreased, opposite to the trend in diet. However, the correlation between penguin diet and minke whale presence, similar to that observed at other nearby penguin colonies^5,21^, may indicate a different form of competition, interference competition, from the minke whales that can consume entire, small krill aggregations in one multi-lunge dive^67^.

The overall decrease in silverfish aggregation abundance during the study period confounds the foraging picture, and more study will be needed to establish the seasonal patterns of silverfish in the McMurdo Sound food web. The decrease of silverfish detections could be due to predation pressure by Weddell seals^68^, which along with emperor penguins are abundant initially at the ice edge (feeding on fish, penguins feeding on krill) but which then moved to the fast ice interior^38,39^ with the arrival of killer whales (both mammal- and fish-eating types). Overall, the abundance of silverfish then became lower beneath the fast ice interior, where the seals had moved (emperor penguins exited the area to molt). It is also possible that since our acoustic detection was limited to aggregations, silverfish may have dispersed, and even ascended, to prey upon the increasing abundance of krill, and thereby became more available to penguins.

## Conclusions

Overall, results indicated that the McMurdo Sound Polynya MIZ constituted a trophic hotspot, where high-quality prey patches resulted in concentrated predation^23,37,48^. Adélie penguins, as an easily-studied indicator species of food web status^69^, foraged preferentially along or within several kilometers of the fast ice edge (Figs. 2, 3), and followed the ice edge retreat southward, as did the minke whales^41^. Our work supports the idea that elevated trophic transfer occurs in the many examples of MIZs that exhibit high abundances of predators^12,23,26,50^, and warrants special inclusion of MIZ metrics and feedbacks into ecosystem assessments and models. Despite what appear to be optimal foraging conditions for both predators (high prey availability) and their mid-trophic prey (high levels of phytoplankton), the distribution of krill at the fast ice edge agrees with the expected response to predation pressure, and is indicative of behaviorally-mediated trophic structuring in the McMurdo Sound Polynya MIZ. Such top-down controls could limit trophic transfer in a situation where prey are not saturated^5,21^, and could cause diet shifts among predators competing for the same prey. Understanding these dynamics can inform food web models that currently lack competition^70,71^, and allow management decisions that more appropriately consider ecosystem function in addition to species’ life histories and abundance. Though a termination time span exists, for now, the recently designated Ross Sea Region Marine Protected Area includes within its boundaries the MIZs of the McMurdo Sound, Terra Nova Bay, Ross Sea, and Ross Passage/Pennel Bank polynyas. As demonstrated herein (also^5,26,50^), these are likely the hottest biological hotspots in the region, and confirm that the Antarctic treaty powers made a wise decision^72^.

## Methods

### Ethics statement

All penguin survey, capture and handling methods performed during data collection for this study were approved under Antarctic Conservation Act (ACA) permits (#2006–010, 2011–002), issued by the U.S. National Science Foundation and the U.S. Antarctic Program; and Institutional Animal Care and Use Committee permits issued by Oregon State University’s (ACUP # 3672, 4130). Studies did not involve endangered species. All data collection activity and field study was performed in accordance with these permits and additional relevant ACA guidelines and regulations.

### Study area

We conducted this study, from early December 2014 through late January 2015, in the outer fast ice region of McMurdo Sound (Fig. 1). McMurdo Sound is especially suited to further investigation of a mesoscale MIZ preyscape, due to its physical attributes and the logistical support available. The Sound is a U-shaped, southern extension of the Ross Sea, 55 × 55 km, >3000 km^2^ in area and >700 m deep (Fig. 1). For a large part of the year, its southern half is covered by fast ice (sea ice attached to land, Ross Island to the east, McMurdo Ice Shelf to the south and Victoria Land to the west), with the ice edge established in winter almost always occurring at the same location adjacent to Cape Royds, and extending westward^54^. The northern portion of the Sound is occupied by the McMurdo Sound Polynya, which is a latent heat polynya kept open by prevailing southerly wind continuously advecting sea ice^8^. Depending on wind strength and direction, the polynya may be completely open, or may be covered by various concentrations of pack ice.

Lacking an oceanographic research vessel, we investigated a marine preyscape using tracked vehicles to access a sampling grid set up on and through the McMurdo Sound fast ice, driving out from McMurdo Station in the southern Sound (Fig. 1). In order to compare a preyscape relatively insulated from predation (fast ice interior) with one in which predators are abundant (ice edge), the sampling grid was composed of 25 cm diameter holes drilled through the fast ice, from its interior out to its edge. Through these holes we repeatedly deployed an acoustic- and flourometric-equipped remotely-operated vehicle (ROV). We made three passes covering the entire sampling grid: pass 1, 16 November-2 December; pass 2, 3–19 December; pass 3, 20 December-7 January. Up to four stations were completed each day.

Sea ice cover was monitored using MODIS visible-band imagery (from the 250 m resolution Corrected Reflectance[True Color] layers of the NASA Worldview website (http://worldview.earthdata.nasa.gov/; downloaded for 2014–2015); as well as daily records kept at our camp at Cape Royds. The distance from foraging locations to the fast ice edge was found as the minimum distance to the ice edge upon a particular date^54^. (Changes to the ice edge occurred 3 times that we could distinguish from satellite images during the study period; Fig. 5).

### Preyscape and water column data acquisition

To assess the temporal and spatial variability of the preyscape, we sampled the grid (Fig. 1) three times with an acoustic-capable ROV (SCINI)^73,74^. Ice edge stations were located within ca. 50 m of the ice edge during Pass 1, prior to ice edge retreat. The remaining stations were 1 km apart along three transects from the edge into the fast ice interior; along the edge stretching for 30 km, stations were 3 km apart. We report on the latter two of these passes (grid samplings) that overlap with penguin biologging and whale abundance surveys. We began to deploy data loggers on the foraging adult penguins after 20 December, as by that time, chicks had hatched and penguins were foraging much more frequently than earlier in the study. Details of biologging are provided below.

Krill and fish were sampled acoustically and visually using the tethered SCINI ROV^66^. SCINI contained cameras and thrusters, and towed a sensor package consisting of a WET Labs fluorometer (ECO-AFL/FL) and a single-beam Biosonics 120 kHz DT-X echosounder^74^. The echosounder operated at a nominal ping rate of 1 ping s^−1^; however, this rate was occasionally adjusted if false bottom signals were observed. The general profile of a dive included a surface transect of ~300 m horizontal distance, where the acoustic transducer faced downward, and also a dive to ~120 m if conditions allowed. Our surveys characterized the surface 200 m because the signal-to-noise ratio of the transducer allowed resolving −80 dB targets at ~ 100 m range.

Raw acoustic data were analyzed using Echoview software^75^. Echogram data were saved to a depth of 500 m, and background noise was removed^76^. All acoustic aggregations >4 pings wide were manually delineated, and acoustic energy of the aggregations was integrated into bins of 6 s wide by 1 m in depth. To ground truth acoustic signals, coincident visual targets were identified to the lowest taxonomic classification possible. Where visual targets were not identifiable, acoustics were verified by comparing aggregation target strength, shape, density, and texture and depth to a set of aggregations with positive visual classification.

Acoustic returns are presented as integrated acoustic energy (volume backscattering strength[Sv], in units of dB re m^−1^). We did not attempt to transform integrated acoustic energy into biomass, since there was not enough size class information to construct acoustic models of identified organisms. Therefore, with the assumption that krill and silverfish body size distributions did not change substantially over the 8-week sampling period, the acoustic values reported are comparable within the classified groups of krill or silverfish, but not between these two groups.

Water column properties were measured at each ROV sampling station using a Seabird Electronics 19+ CTD that measured salinity, temperature and fluorescence. The depth of the 1% light level penetrating into the water column was calculated using the depth-binned chlorophyll concentrations fed into an optical model^77^ that utilizes the thicknesses and types of snow, sea ice, and water column properties to calculate underwater irradiances.

### Adélie penguin diet and foraging

We assessed the overall diet being consumed by penguins in the Cape Royds colony by observing the color and consistency of food as it passed from parents to chicks during feeds observed on a daily basis; this was easily seen by using binoculars for a side view of feeding events^5,21,46^. The percentage of krill, fish and “other” was estimated in each observation. Previous ground-truthing, by stomach pumping, revealed that entirely pink paste was crystal krill (100%), and gray, chunky paste was silverfish (100%)^21,42,46,47^. Previous work also showed that chick diet represented quite well that of the adults/parents^42,43^. Diet observations began on 14 December 2014 (when approximately 5% of chicks had hatched) and continued until 22 January 2015. To obtain adequate sample size, diet observations were grouped into 5–6 d periods, centered to coincide with the start of the second pass of prey sampling (i.e., 20 December; see below).

Once sufficient numbers of chicks hatched thus to make their parents easily available (20 December), we equipped randomly 18 selected parents with SPLASH tags (wildlifecomputers.com). Parents with chicks forage intensely, and are also easily located upon return to the colony. The tags recorded depth, light, and temperature each second, and determined position using ARGOS satellites (see below). The tags, applied to the lower back just forward of the tail, weighed 62 g (1.6% of a 4-kg Adélie penguin) and had a cross-sectional area of 3.2 cm^2^ (1.0–1.6% of a penguin’s cross-sectional area). We attempted to retrieve the tags after one or two foraging trips (this was not always possible) and attach them, using tape, to the next set of individuals^78^. Only one parent was instrumented from a particular nest. Up to three penguins were instrumented at a given time; penguins recorded from 1–7 foraging trips per deployment.

The tags were set to transmit locations every 45 s for the first eight successive transmissions and then switched to once every 90 s, with up to 1440 transmissions allowed per day. They turned off if they were dry for 6 h (penguin out of water) in order to conserve batteries. Diving data were downloaded from the tags after retrieval. We used data from all trips for which both dive data and location data were available, except for cases in which locations indicated errors^5,48,79^. Because of different temporal resolution between dive (1 s) and accepted location data (15 min, after interpolation), we used temporal proximity to assign each dive an approximate geographic location, as we have done previously^5,62^. We assumed that the follow-on interpolated positions were adjacent in space and time from a temporally known position (TKP). We removed highly interpolated positions (>30 min TKP), which allowed more dives to be included in the analysis, while reducing the locational errors. In total, the data set included information from 18 tagged penguins making 50 foraging trips.

Dive data were initially processed using the program divesum (v.7.5.5; G. Ballard, unpublished software). This program computed several dive parameters, such as maximum dive depth, time spent at the bottom phase of each dive, and number of undulations (changes in depth >1 m, from ascent to descent). Divesum also classified dives into three types: foraging, exploratory, and other^5,48,79^. Foraging dives contain undulations in the bottom phase, with undulations considered to represent prey capture attempts; exploratory dives were just up and down with no time spent at depth and “other” were shallow, traveling dives^62,80^. Divesum also summarized light recorded during foraging dives into means of light levels at ~ 5 m depth (indicating surface light availability) and light levels during the dive bottom phase (indicating light levels during foraging). Light levels were log-transformed before performing statistical tests^81^.

### Predator/competitor abundance

To provide more ecological context, we investigated the prevalence of various potential penguin competitors. Due to inability to assess presence of these species in the open waters of the polynyas, we assessed abundance along the ice edge as an index to prevalence. The occurrence of cetaceans in McMurdo Sound for several years, including the study year, has been detailed in publications elsewhere^20,41^. We obtained an index to whale presence by visual observations (telescope and binoculars) once or twice per day, weather permitting, from a coastal hill at Cape Royds 30 m above sea level^20,21^. Observation periods lasted for an hour. We could effectively scan along the ice edge out to about 3 km, as well as adjacent open or pack-ice-covered waters. Observations were made between 20 November and 26 January, with gaps during 22–26 December, 29 December-9 January, and 19–25 January, in part related to poor visibility.

To ground-truth the abundance of whales, and assess abundance of seals and emperor penguins along the entire cross-Sound ice edge, we counted these species by helicopter at several-day intervals, mid-November to mid-January, weather permitting. Flights were made 20 November, 2, 8 and 27 December, and 10 January. The flights followed the fast ice edge in both directions between Cape Royds as well as a few kilometers north, i.e. both arms of the fast ice “L” (Fig. 1;^54^).

### Statistical analysis

Penguin dive data were tested for a relationship to the fast ice edge using regression analysis in the R computing environment^82^, controlling for individual penguin as a random effect using the “lmer” function within the {lme4} package as part of the maximum likelihood method (REML)^83^. To test for relationships or correlations among penguin dive parameters, mixed-effect models were evaluated with the distance to the fast ice edge as the single fixed-effect, and individual penguin as a random effect. To test for sinusoidal variability in penguin dive parameters, similar models were constructed predicting dive parameters (treatment) using a cosine function with wavelength of one day and phase shift of pi/6 (i.e. the peak of the cosine function set to 14:00 daily) as the fixed effect and individual penguin as a random effect. Individual tests were performed for each fixed-effect. Light-related variables and the distance of foraging penguins to the fast ice edge were log-transformed to normalize their distributions.

Kendall rank correlations were calculated between penguin diet fractions and preyscape variables, with multiple tests accounted for with Holm-Bonferroni corrections (also using R). We assumed that penguin diet observations and dive data were representative of the colony population as a whole. This approximation is acceptable given that individual penguins did not forage in completely independent areas^37,48^, and diet observations were randomized as opportunistic encounters.

To test for difference in prey distribution at the ice edge, volume backscattering values were pooled into edge (still beneath fast ice but within 1 km of open water, the likely excursion distance of a minke whale foraging under the ice^41^; see Fig. 1) and interior (>1 km under the fast ice edge). We then binned the acoustic returns into 10 m-depth intervals, and tested whether krill abundance differed, across depths, between edge sites – where penguins (and minke whales) could access krill – and interior sites under the ice – where penguins (and minke whales) could not access krill. Across deployments, abundance measurements were typically zero with occasional aggregations of krill or silverfish. Where krill occurred, they usually spanned multiple depths, thus krill abundance was not independent across depths for a given date and site of deployment. The patchiness of krill aggregations meant that hypothesis testing with typical parametric tests would have violated test assumptions.

Thus, we performed a permutation test^84^. For each permutation, the designation of ice edge versus interior was reassigned keeping the number of edge and interior deployments constant at 30 and 54. The difference in mean krill abundance between edge versus interior sites, *x*_*j*_, was recorded for each of *B* = 10,000 permutations. The *p*-value for observing the recorded difference, *X* = [depth at edge] –[depth at interior sites], was calculated using the following equation^82^:

$$P=\frac{1+{\sum }_{j=1}^{B}I({x}_{j}\le X)}{B+1}$$

where *I* is the indicator function that takes the value one when the argument is true and zero when it is false. Note that for depths 10–60 m (the standard foraging depth range of Adélie penguins) we expect that *x*_*j*_ < *X* for prey species, which would be indicative of lower prey concentrations at edge sites.

### Graphics and plotting

Figures 1 and 5 were produced using QGIS^85^ geographic information system software. Data used for coastlines and glacial ice are derived from the SCAR Antarctic Digital Database^86^ available through the QAntarctica^87^ package, and bathymetry data is from Antarctica New Zealand and GNS Science^88^. Figures 3, 4, 6, and 7 were producing using the Matplotlib^89^ graphical package, with Fig. 6 using the additional seaborn^90^ package. Figures 2, 8, 9, and 10 were made using Microsoft Office 2016.

## Data Availability

Except where otherwise noted in the text, all data for this paper are hosted at BCO-DMO, in project ‘Food web dynamics in an intact ecosystem: the role of top predators in McMurdo Sound’ (http://www.bco-dmo.org/project/665131). Data on penguin foraging and whale censuses are available at California Avian Data Center (CADC) hosted by Point Blue Conservation Science and metadata are registered with the “Antarctic Master Directory” (http://gcmd.nasa.gov/KeywordSearch/Home.do?Portal=amd&MetadataType=0). Data are and will be available at CADC: http://data.prbo.org/apps/penguinscience/.
